# The Influence of Chimeric Antigen Receptor Structural Domains on Clinical Outcomes and Associated Toxicities

**DOI:** 10.3390/cancers13010038

**Published:** 2020-12-25

**Authors:** Ashleigh S. Davey, Matthew E. Call, Melissa J. Call

**Affiliations:** 1 Structural Biology Division, The Walter and Eliza Hall Institute of Medical Research, Parkville 5052, Australia; mecall@wehi.edu.au; 2 Department of Medical Biology, The University of Melbourne, Parkville 3052, Australia

**Keywords:** CAR-T cell therapy, CD19, clinical trials, CRS, toxicity

## Abstract

**Simple Summary:**

The development and refinement chimeric antigen receptor (CAR)-T cell immunotherapy has significantly improved the prognosis of patients with B cell malignancies. Severe treatment related toxicities however remain a significant challenge for the field. This perspective reviews 17 clinical trials of the most widely used anti-CD19 (FMC63) CAR-T cell therapies, with the aim of dissecting the contribution of the structural and costimulatory domains of the CAR to clinical outcomes and toxicities. The CD28 structural hinge and transmembrane CAR domains are highlighted as strongly associated with both clinical efficacy and severe toxicity. This perspective supports further investigation into the structural CAR domains for improved CAR design and safer CAR-T cell therapies.

**Abstract:**

Chimeric antigen receptor (CAR)-T cell therapy has transformed the treatment of B cell malignancies, improving patient survival and long-term remission. Nonetheless, over 50% of patients experience severe treatment-related toxicities including cytokine release syndrome (CRS) and neurotoxicity. Differences in severity of toxic side-effects among anti-CD19 CARs suggest that the choice of costimulatory domain makes a significant contribution to toxicity, but comparisons are complicated by additional differences in the hinge and transmembrane (TM) domains of the most commonly used CARs in the clinic, segments that have long been considered to perform purely structural roles. In this perspective, we examine clinical and preclinical data for anti-CD19 CARs with identical antigen-binding (FMC63) and signalling (CD3ζ) domains to unravel the contributions of different hinge-TM and costimulatory domains. Analysis of clinical trials highlights an association of the CD28 hinge-TM with higher incidence of CRS and neurotoxicity than the corresponding sequences from CD8, regardless of whether the CD28 or the 4-1BB costimulatory domain is used. The few preclinical studies that have systematically varied these domains similarly support a strong and independent role for the CD28 hinge-TM sequence in high cytokine production. These observations highlight the value that a comprehensive and systematic interrogation of each of these structural domains could provide toward developing fundamental principles for rational design of safer CAR-T cell therapies.

## 1. Introduction

Recent decades have seen the development of groundbreaking new treatments for cancer. The emergence of adoptive cellular therapies, including chimeric antigen receptor (CAR)-T cells, represents an advance that has dramatically improved upon classical cytotoxic chemotherapies and led to significant improvements in patient outcomes for advanced and refractory B cell malignancies. CAR-T cell therapy utilises engineered receptors (CARs) to redirect a patient’s cytotoxic T cells to a targeted cancer antigen of choice. This therapy is a multi-step process involving leukapheresis of a patient’s blood, the genetic modification of their T cells to express a tumour-antigen-specific CAR, followed by ex vivo expansion and re-infusion of the CAR-T cells back to the patient where they selectively seek out and kill target tumour cells. Following high remission rates in clinical trials, two CD19 CAR-T cell products were approved by the FDA in late 2017 for the treatment of refractory non-Hodgkin lymphoma (NHL) and for patients with relapsed or refractory B cell acute lymphoblastic leukemia (B-ALL) up to the age of 25 [1]. There are now over 200 CAR-T cell clinical trials underway for a range of cancer antigens (clinicaltrials.gov). However, safety concerns and general applicability to many tumour types are issues that still need to be resolved. A great deal of research is now underway to modulate specific properties of CAR-T cells with the aim of improving their safety and efficacy for a wider range of cancers. In this perspective, we examine the influence of CAR domain structure on the clinical outcomes and toxicities observed in the most extensively studied CAR-T cell therapies, the anti-CD19 CAR-T cells using the antibody single-chain variable fragment (scFv) FMC63. While differences in efficacy and safety profiles between the two FDA-approved anti-CD19 therapies may reasonably be ascribed primarily to their use of different costimulatory signalling domains in the CAR constructs, we highlight clinical and preclinical observations suggesting that the hinge and transmembrane (TM) domains, long considered to be functionally inert structural features, make surprisingly strong contributions to CAR-T cell toxicity. Understanding the mechanisms underlying these effects will be a key step in improving rational CAR design.

### 1.1. Components of the Chimeric Antigen Receptor (CAR)

Naïve T cells require dual signalling interactions for activation, first between the multi-chain T cell receptor (TCR) complex and peptides presented by an interacting cell’s MHC proteins (signal 1), and additionally from costimulatory receptor:ligand interactions (signal 2). The first generation of CARs generated by Esshar and colleagues utilised the antigen specificity of an scFv fused to the entire CD3ζ protein to mimic signal 1 in T cell activation [2]. The resulting CAR was expressed well at the cell surface and was successful in redirecting T cells to target tumour antigens. However, CAR-T cell persistence was poor, and cells showed signs of exhaustion prior to tumour clearance [3,4,5,6]. This prompted the development of second-generation CARs which also contain an scFv antigen recognition domain and intracellular CD3ζ signalling tail, but additionally incorporated a flexible hinge domain for improved antigen reach and a costimulatory domain to provide signal 2 required for strong and durable T cell activation [7]. The intracellular costimulatory and signalling domains are connected to the extracellular scFv and hinge via a TM domain, which is often an extension of the costimulatory or hinge sequence. Most CAR constructs exist as dimers on the cell surface, stabilized by disulfide bonds between hinge domains and inter-monomer TM interactions [8]. Inclusion of the additional domains provided second-generation CAR-T cells with the requisite signals for activation and significantly improved persistence and clinical outcomes in treated patients [9,10,11]. Further iterations of CARs continue to be developed, including those with two costimulatory domains (third-generation CARs) as well as CARs with inducible expression of a transgene product such as cytokines (fourth-generation CARs/TRuCs) [12,13,14].

### 1.2. Limitations of CAR-T Cell Therapy

Since the development and initial success of second-generation CAR-T cell therapies in the clinic, many groups have now shifted focus to manipulating specific domains of the CAR with the aim of improving target cell recognition and therapeutic efficacy, and limiting CAR-associated toxicities. A significant leap in the field came with the introduction of a costimulatory domain into the CAR which significantly improved the persistence and efficacy of clinical CAR-T cell products [7]. The choice of which specific costimulatory domain is more clinically effective though is not yet unanimous. Extensive work is underway to further improve upon these current second-generation CAR-T cell constructs by altering specific CAR domains including the hinge, TM and costimulatory domains, particularly for the treatment of more difficult immunosuppressive solid tumours [8,15,16,17,18,19,20,21,22].

Clinical trials of CAR-T cell therapies all report similar treatment-related toxicities, including cytokine release syndrome (CRS) and neurotoxicity. CRS is triggered by the rapid systemic release of inflammatory cytokines including IFNγ, IL-6 and IL-1 into the blood by activated CAR-T cells and endogenous myeloid cells [23,24,25]. The symptoms of CRS in response to CAR-T cell therapy range from mild flu-like symptoms to severe symptoms such as hypotension, organ toxicity and acute respiratory distress, which can collectively be life threatening and often result in patients spending extended periods of time in the intensive care unit (ICU) [26]. The second major toxicity reported following CD19-targeted CAR-T cell therapy is neurotoxicity. Similar to CRS, neurotoxicity symptoms also range in severity and type, including headaches, delirium, dysphasia, ataxia, dysmetria, decrease in level of consciousness, seizures and acute cerebral edema [27,28,29]. The incidence of neurotoxicity among CAR-T cell-treated patients is quite variable, with some neurological events occurring in combination with CRS symptoms and others at different times or in the absence of CRS, suggesting that at least in some cases the mechanism of CAR-T cell induced neurotoxicity is different to that of CRS [30,31]. Comprehensive and timely management plans are also in place for the treatment of neurological toxicities, often involving the administration of systemic corticosteroids including dexamethasone, which has the unfortunate consequence of interfering with CAR-T cell function [32]. While both CRS and neurotoxicity symptoms can be treated in the clinic, many patients require expensive ICU administration and support, thus prevention of such severe toxicities with CAR-T cell treatment is ideal for further progression of this promising therapeutic.

It has generally been presumed that the costimulatory domains are mostly responsible for the severe toxicities observed with CAR-T cell therapies due to the increased potency and persistence observed in second-generation CARs compared to first-generation CARs [3,4,5,6]. Considering many of the costimulatory domains used clinically extend from the intracellular domain though the TM to the extracellular hinge and scFv, it is difficult to discern the contribution of the TM and hinge structural domains individually to clinical efficacy and toxicity development. We therefore examined clinical trials of the most extensively published anti-CD19 FMC63 scFv CARs, all of which contain a CD3ζ signalling tail, to understand whether CAR-related toxicities are defined by the costimulatory domain alone or whether structural features such as the hinge and TM domains of the CAR also play a role.

## 2. Clinical Trials of Anti-CD19 CAR-T Cell Therapy

CD19 is a cell-surface glycoprotein uniformly expressed during all stages of B cell differentiation and is present in more than 95% of B cell malignancies [33]. Under normal physiological conditions, CD19 acts as a co-receptor in complex with the B cell receptor and other surface markers to modulate downstream signalling pathways that induce B cell proliferation and activation [34]. Due to its homogenous and high expression in many B cell malignancies and the fact that bystander B cell depletion can be managed clinically, CD19 has become an attractive target for the initial testing and translation of CAR-T cell immunotherapy, evident by over half of all CAR-T cell clinical trials targeting the CD19 antigen (clinicaltrials.gov). To dissect the clinical implications of individual hinge, TM and costimulatory domains, we collected and summarised data from anti-CD19 CAR-T cell clinical trials in which the CAR construct contained the same FMC63 scFv [35] and CD3ζ signalling tail (Table 1). Clinical trials were included if they used a second-generation CAR containing these domains and provided details of clinical response rate, CRS and neurotoxicity.

**Figure 1:**
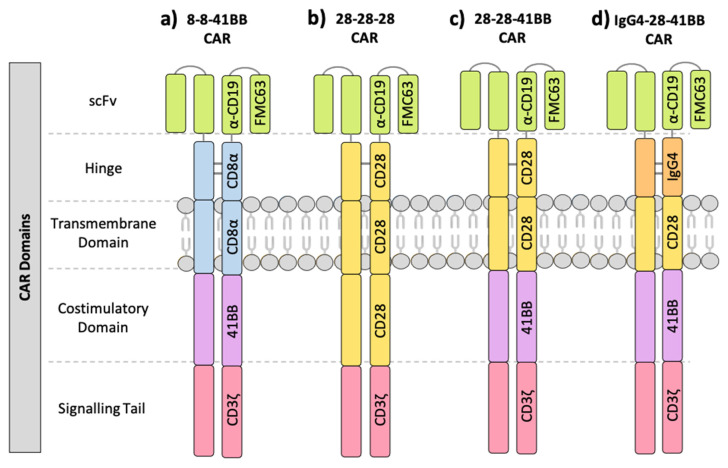
Structural domains of our main FMC63 anti-CD19 CARs. The structural design of the (**a**) 8-8-41BB CARs, (**b**) 28-28-28 CARs, (**c**) 28-28-41BB CARs and (**d**) IgG4-28-41BB CARs reviewed here. The individual scFv, hinge, transmembrane domain, costimulatory domain and signalling tails are labelled. Horizontal lines between hinge domains indicate disulfide bonds.

The majority of FMC63 CAR-T cell clinical trials have been conducted on patients with B-ALL and NHLs including diffuse large B cell lymphoma (DLBCL), follicular lymphoma (FL), mantle cell lymphoma (MCL) and marginal zone lymphoma (MZL), due to their generally high and stable expression of CD19 (Table 1). These CD19 CARs generally adopt one of two multi-domain structures: 1. FMC63 scFv, CD8α hinge and TM, 41BB costimulatory domain and CD3ζ signalling tail (8-8-41BB CAR; Figure 1a), or 2. FMC63 scFv, CD28 hinge, TM and costimulatory domain, and CD3ζ signalling tail (28-28-28 CAR; Figure 1b). There is also one published clinical trial utilising a CAR with a CD28 hinge and TM but a 41BB costimulatory domain (28-28-41BB; Figure 1c) [36], and another with an IgG4 hinge, CD28 TM and 41BB costimulatory domain (IgG4-28-41BB; Figure 1d) [37,38]. In the clinical trials reviewed here, all CD28 costimulatory domain-containing CAR-T cells were transduced using gamma retrovirus, while all 41BB-containing CAR-T cells were transduced using lentivirus (Table 1). The dosage of CAR-T cells within and between clinical trials ranges significantly from 3 × 10^4^ cells to 3.7 × 10^8^ cells per kg (Table 1). By directly comparing the clinical trials containing each of these four iterations of the FMC63 CAR, trends towards particular structural domains that influence specific outcomes such as response rate and toxicity may be exposed.

### 2.1. The Effect of CAR Domains on Clinical Response

**Figure 2:**
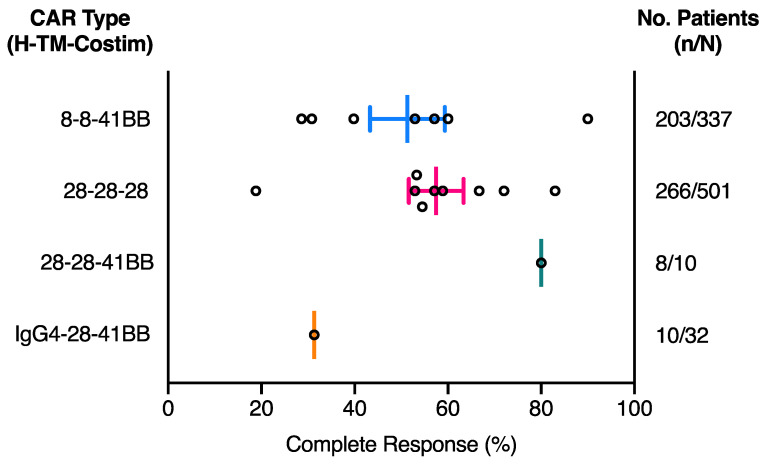
Effect of CAR composition on complete response in patients treated with CD19 CAR-T cell therapy. CAR type separated based on hinge (H), transmembrane (TM) and costimulatory (Costim) domain composition. All CAR constructs use the FMC63 anti-CD19 scFv and the CD3ζ-chain activation domain. Each circle represents an individual clinical trial listed in Table 1. Complete response (CR) rate calculated from the number of patients who reached a CR at any stage post CAR-T cell infusion (n) as a percentage of all patients treated with the CAR-T cell therapy (N) (Table S1). Middle line represents the mean CR and error bars represent the SEM.

Multiple factors have been reported to impact CD19 CAR-T cell therapy clinical outcome in patients, including whether a patient has received conditioning lymphodepletion therapy prior to CAR-T cell infusion, whether CAR-T cells were cultured with IL-2 prior to infusion and the extent of CAR-T cell persistence in patients [52,53]. For the studies shown in Table 1, we saw no obvious effect of age, conditioning lymphodepletion treatment or CAR-T cell dosage on patient complete response rate (Table 2). Not surprisingly, although the tumour target antigen was identical in all FMC63 CAR-T cell treatments, patient complete response rate does appear to vary depending on tumour type, with B-ALL patients gaining the highest response rates, followed by FL, DLBCL and lastly MCL (Table 2). Considering the limited number of FMC63 anti-CD19 CAR-T cell clinical trials with published results, all clinical trials in Table 1, including those with MCL patients, were included for response and toxicity CAR comparisons. The complete response (CR) rate among patients treated with the various CD19 CAR-T cell therapies was calculated as the number of patients who achieved a CR at any stage post CAR-T cell infusion as a percentage of the total number of patients treated with the CAR-T cell therapy in a single clinical trial (Figure 2). Details of the individual trial patient numbers and other reported patient responses for each clinical trial are detailed in Table S1.

Overall, 28-28-28 CARs and 8-8-41BB CARs have very similar CR rates (Figure 2). Interestingly, in a single study where B-ALL patients were treated with a 41BB CAR that used the CD28 hinge-TM domain instead of CD8α (28-28-41BB CAR), a higher CR rate was observed in comparison to the average for studies using 28-28-28 and 8-8-41BB CARs (Figure 2). Although this is only a single clinical trial of 10 patients, it highlights the 41BB costimulatory domain may be associated with improved clinical response [36]. Furthermore, comparison of this CAR with one in which only the hinge was altered, using IgG4 instead of CD28 (IgG4-28-41BB CAR), a much lower CR rate was observed [37,38]. While the IgG4-28-41BB CAR clinical trial includes a number of participants with the poorest responding MCL tumour type, it highlights the possibility that inclusion of the CD28 hinge-TM structural domains in clinical CAR constructs may also be correlated with higher complete response rate in patients. Further preclinical comparisons and clinical studies are required to investigate the full impact of the individual hinge-TM and costimulatory domain substitutions in isolation and their correlation with response rates.

### 2.2. CRS Toxicity Correlates with CAR Domain Design

There is currently no clear consensus on a CRS grading system for CAR-T cell therapies among institutions worldwide, making CRS severity analysis between CAR-T cell products and trials exceedingly difficult. This is largely due to the fact that the timing and definition of CRS symptoms observed following cellular therapies such as CAR-T cells differs significantly to previously described CRS responses to antibody and drug therapies [54,55,56]. CRS following CAR-T cell treatment was initially graded using the National Cancer Institute Common Terminology Criteria for Adverse Events (CTCAE) grading scale for adverse event reporting [57,58]. However, this scale was developed before cell therapy-induced CRS was well understood. CTCAE was more consistent with an acute cytokine storm typically observed within minutes to hours of antibody or drug treatment but did not take into consideration the more likely delayed onset of CRS symptoms up to a few days post CAR-T cell infusion [57,58,59]. This grading system was modified by Lee and colleagues in 2014 to more accurately define mild, moderate, severe, and life-threatening CRS symptoms regardless of the inciting agent [60]. This guideline also included treatment recommendations for each of the CRS grades described and has been consequently used by many clinical trials conducted after this date. An alternative CRS grading system was developed and used for CTL-019 CAR T cell trials from the University of Pennsylvania (Penn Grading Scale), which had a similar grading structure to the 2014 Lee et al. scale but did not use absolute cut-off values for vital signs such as % oxygen requirement to define CRS grade, as such values can vary in severity depending on the individual patient [41]. The Penn Grading System was also designed in a way that allowed for CRS symptoms to be reproducibly graded among different institutions, CAR-T cell target antigens and disease settings. While we do not provide a detailed comparison of the various CRS grading methods here, it is clear that a consensus on an accurate CRS grading program and prompt management plan for CAR-T cell induced CRS symptoms that is used worldwide in all clinical trials would be more beneficial for inter-trial analysis [61].

**Figure 3:**
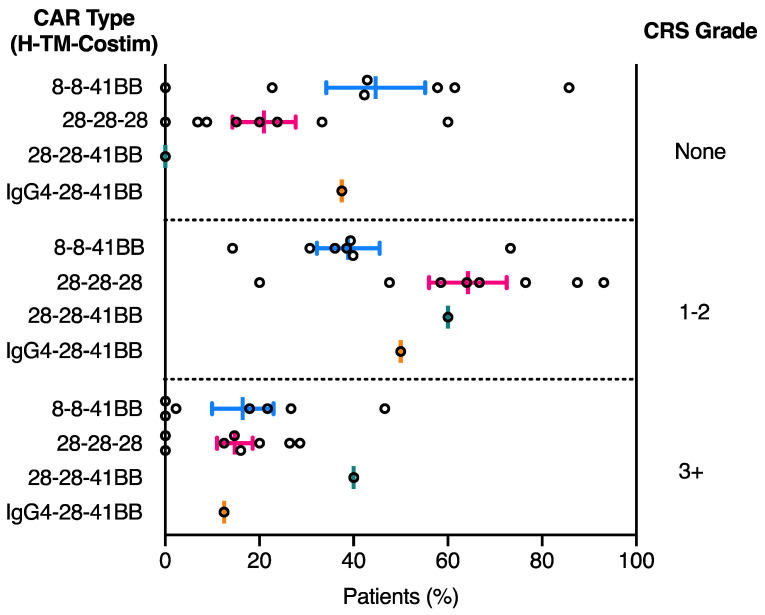
CAR domain structure influences CRS severity in patients treated with CD19 CAR-T cell therapies. Both CAR type, based on hinge (H), transmembrane (TM) and costimulatory (Costim) domain composition, and CRS grade, separated into none, grade 1–2 and grade 3+, are represented on the left and right *y* axis, respectively. Each circle represents the percentage of patients in a single clinical trial listed in Table 1 that developed the corresponding grade of CRS (none, grade 1–2 and grade 3+). Middle line represents the mean percentage of patients with the corresponding grade of CRS and error bars represent the SEM (Table S2).

Despite the above limitations, we felt that the CRS grading systems used in many of the FMC63 CAR-T cell clinical trials were similar enough to yield informative comparisons among the different CAR structural designs (Table 3). To assess the effect of domain structure on CRS development, patient CRS grade was collected for each clinical trial presented in Table 1 and taken as a percentage of total patients within each clinical trial (Table S2). The percentage CRS development for each grade and clinical trial was subsequently plotted by CAR domain configuration to examine correlations between CRS diagnosis and CAR domain structure. Overall, the incidence of CRS in patients treated with CAR-T cell therapy is high, with 60–80% of patients developing some level of identifiable symptoms. While the mean percentage of patients in the highest category of CRS (grade 3+) does not differ between CAR-T cell treatment products, a clear correlation between CRS development and CAR domain configuration is observed for grade 1–2 CRS (Figure 3). The 28-28-28 CAR-T cell treatment resulted in a higher proportion of patients developing CRS compared to 8-8-41BB CAR-T cell treatment (Figure 3). It is difficult to discern the contribution of the CD28 costimulatory domain to this increased CRS toxicity, as 28-28-28 and 28-28-41BB CAR-T cell-treated patients show similar levels of grade 1–2 CRS development. However, the 28-28-41BB CAR-T cell therapy does emerge with a higher incidence of more severe grade 3+ CRS in comparison (Figure 3). Interestingly, the use of CD28 hinge and TM domains in a 41BB CAR (28-28-41BB) was associated with an increased proportion of patients developing CRS, rising to a level similar to that observed in the 28-28-28 CAR (Figure 3) [36]. While this is only a single clinical trial, it suggests that CRS severity is at least in part attributable to the CD28 hinge and TM domains independently of which costimulatory domain is used. The same degree of increase in CRS was not observed in the trial using an IgG4-CD28 hinge-TM domain configuration (IgG4-28-41BB), further underscoring that the identity and/or combination of hinge-TM structural domains can exert significant influence on manifestation of CRS toxicity even when the scFv, costimulatory and activation domains are the same (Figure 3) [37,38]. Additional research and clinical trials that make direct comparisons among CAR configurations will be required to identify the relative contributions of each individual CAR domain in developing CRS.

### 2.3. Relationship between CAR Domain Structure and Neurotoxicity Development

Neurotoxicity has been reported to occur both concurrently and independently of CRS toxicity in anti-CD19 CAR-T cell clinical trials. In general, neurotoxicity events that occur concurrently with CRS are of a shorter duration and severity (grade 1–2), while delayed neurotoxicity occurring post-CRS can arise up to 3-4 weeks after CAR-T cell therapy and is more commonly associated with grade 3+ neurotoxicity [37,38,51,62]. Neurotoxicity severity has been reported to fluctuate rapidly once diagnosed, demonstrating the requirement for close patient monitoring and precise management plans to prevent rare fatal events. Similar to CRS toxicity, there are multiple grading scales that can be used to assess the level of neurotoxicity in patients receiving CAR-T cell therapy. The most common grading protocol used in the clinical trials reviewed here is the National Cancer Institute Common Terminology Criteria for Adverse Events (CTCAE), with CTCAE v3.0 used prior to 2009 [58] and CTCAE v4.0 after 2009 [57] (Table 3). The CTCAE neurotoxicity grading systems have limitations due to the unique characteristics and timing of neurotoxicity symptoms following CAR-T cell treatment compared to antibody and drug treatments. Consequently, a unique condition titled CAR-T cell-related encephalopathy syndrome (CRES) was introduced by the multi-institutional CAR-T cell therapy associated toxicity (CARTOX) working group. The CRES grading system encompasses a 10-point patient questionnaire to capture cognitive and attentive dysfunction combined with clinical tests to assess intracranial pressure and severity of seizures [32]. More recently, the American Society for Transplantation and Cellular Therapy (ASTCT; previously ASBMT) coined the term immune effector cell-associated neurotoxicity syndrome (ICANS) for CAR-T cell related neurotoxicity, using a modified version of the CARTOX screening grading system to also take into account patient consciousness, motor symptoms and cerebral edema symptoms [63]. A retrospective study using these three methods (CTCAE v4.03, CRES and ASTCT) to grade neurotoxicity events post CAR-T cell treatment in the JULIET trial uncovered the overdiagnosis of neurotoxicity using the CTCAE v4.03 grading system (45% of patients) in comparison to both CRES and ASTCT grading systems (both 17.1% of patients). Many of the patients only diagnosed with neurotoxicity using the CTCAE v4.03 and not CRES or ASTCT grading systems had mild symptoms such as headaches which were thought to be non-specific to the CAR-T cell treatment [64]. Clearly there is a disparity in the published grading systems for neurotoxicities seen after CAR-T cell therapy. A refined grading system proposed by 49 CAR-T cell experts and supported by the ASTCT was published in 2018 in the hope that future CAR-T cell clinical trials can use a consensus grading system for more accurate toxicity reporting and inter-trial analysis [63]. Use of such a universal grading system would enable better comparison of clinical trial products and outcomes.

**Figure 4:**
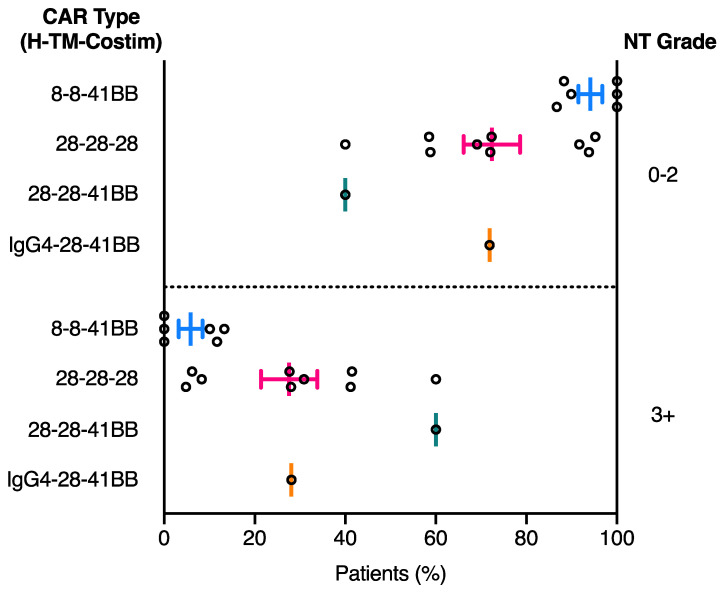
Grade of neurotoxicity development in patients treated with CD19 CAR T cell therapies is related to CAR domain structure composition. CAR domain structure is separated based on hinge (H), transmembrane (TM) and costimulatory (Costim) domain composition on the left *y* axis, while neurotoxicity grade is separated into grade 0–2 or grade 3+ on the right *y* axis. Each circle represents the percentage of patients in a single clinical trial listed in Table 1 that developed the corresponding grade of neurotoxicity (grade 0–2 and grade 3+). Middle line represents the mean percentage of patients with the corresponding grade of neurotoxicity and error bars represent the SEM (Table S3).

Neurotoxicity symptoms were reported and graded in 16 of the 17 clinical trials listed in Table 1 (Table 3). To assess the effect of domain structure on neurotoxicity development, the percentage of patients that developed each grade of neurotoxicity was calculated for each clinical trial presented in Table 1 (Table S3). The percentage neurotoxicity development for each grade and clinical trial was subsequently plotted by CAR domain configuration to examine correlations between neurotoxicity diagnosis and CAR domain structure. Overall a clear increase in severe (grade 3+) neurotoxicity was observed in patients treated with the 28-28-28 CAR compared to the 8-8-41BB CAR (Figure 4). While the 8-8-41BB CAR showed on average the lowest level of severe grade 3+ neurotoxicity, use of the CD28 hinge-TM or IgG4 hinge and CD28 TM in a 41BB CAR (28-28-41BB and IgG4-28-41BB, respectively) was associated with increased incidence of grade 3+ neurotoxicity that was more in line with the 28-28-28 trials (Figure 4) [36,38]. As for CRS, these observations suggest that use of part or all of the CD28 structural domains is associated with higher toxicity. While the most relevant direct comparisons required to confirm these associations have not been made in any single large clinical trial, as outlined below, there is significant preclinical data that offer strong support for the independent contributions of hinge and TM domains to CAR function and toxicity.

## 3. Preclinical Evidence of CAR Domain Design Influencing CAR-Related Toxicities

CRS symptoms are caused by a significant elevation of blood cytokine levels as a result of excessive cytokine secretion by both CAR-T cells and other immune cells. Consequently, CAR T cells which exhibit reduced cytokine secretion to levels that induce less CRS toxicity but still promote effective T cell proliferation and cytotoxicity are required. Optimisation of CAR domain configuration towards this end is a crucial goal, but unravelling the contributions of different domains is complicated by comparisons in which hinge, TM and costimulatory domains are all varied at the same time. A small number of carefully controlled preclinical studies have recently begun to address this problem systematically.

In one recent study focused on modifications to the hinge-TM domains alone, Ying and colleagues [15] generated CTL-019 variant CARs (FMC63 scFv with CD8α hinge and TM domains, 4-1BB costimulatory domain and CD3ζ tail) with small extensions to the CD8α hinge and TM domains. One of the longer hinge-TM constructs (named CD19-41BBz(86) in their study) showed a significant reduction in IFNγ, TNFα, IL-2 and IL-4 production in vitro when co-cultured with human CD19 target cells. Treatment of B cell lymphoma patients in a small phase I clinical trial with the CD19-41BBz(86) CAR-T cell therapy yielded good response rates, low circulating cytokines and little to no development of CRS or neurotoxicity symptoms post-treatment [15]. This study demonstrates that even small changes to the hinge-TM domains of a CAR can significantly impact cytokine production and CRS development. The mechanism by which this extension of only a few amino acids on each end of the hinge-TM yields such significant effects is unknown.

Another study by Alabanza and colleagues [65] directly compared FMC63 anti-CD19 CARs containing the same CD28 costimulatory domains and CD3ζ tail with either CD28 or CD8 hinge-TM regions (named FMC63-28Z and FMC63-CD828Z in their study, respectively). Both CARs showed similar cell-surface expression and induced degranulation upon target cell recognition, however secretion of IFNγ and TNFα was significantly lower in FMC63-CD828Z CAR-T cells compared to FMC63-28Z CAR-T cells. The same hinge-TM substitutions in fully human anti-CD19 (HuCD19) and human VEGFR2 (hVEGFR2) CD28 costimulatory domain-containing CARs also demonstrated reduced cytokine secretion associated with the CD8 hinge-TM region compared to the CD28 hinge-TM region [8,66]. In a separate study examining independent variations of structural and costimulatory domains, Majzner and colleagues [20] showed that both the CD28 hinge-TM and CD28 costimulatory domains contribute to the high cytokine production associated with the FMC63-CD28z configuration compared to the FMC63-CD8-41BBz configuration. Importantly, the CD28 hinge-TM domains conferred improved cytotoxic potency against low-antigen target cells, regardless of the identity of the costimulatory domain, and was associated with both higher potency and increased cytokine production even in first-generation CAR constructs containing no costimulatory domains [20]. Emerging data suggest that such effects of the CD28 hinge-TM domains are attributed to its high propensity to form heterodimers with endogenous CD28 through specific TM sequences within the membrane, thus hijacking native CD28 receptor signalling in addition to CAR-mediated signalling [17,67]. These preclinical observations support the suggestion from our review of clinical trial outcomes that the higher incidence of intermediate CRS and high-grade neurotoxicity observed in trials using FMC63 CARs with CD28-derived hinge-TM and costimulatory sequences may be more closely associated with the structural domains than the costimulatory domain.

## 4. Conclusions

Here we examined FMC63 anti-CD19 CAR-T cell clinical trials in an attempt to unravel the impact of the hinge, TM and costimulatory domains of the CAR protein on patient clinical outcome and toxicities. CD28 hinge-TM containing CARs were associated with a slightly higher average clinical response rate but were also associated with more severe toxicity compared to CD8 hinge-TM containing CARs. While the number of patients and clinical trials contributing to this observation are limited, several recent preclinical studies highlighted above support this association, with CD8 hinge-TM CAR-T cells consistently shown to produce lower levels of cytokines than otherwise identical CD28 hinge-TM CAR-T cells. These studies contribute to an increasing recognition within the field that the hinge-TM structural domains are not functionally inert, and work to unravel the contribution of the hinge and TM domains independently is ongoing. It remains unclear whether one or both of these structural domains contributes directly to these effects, and the mechanisms underlying these functional outcomes are still not well understood. We suggest that a broad systematic interrogation of hinge and TM domain effects on CAR structure, stability, expression levels, signalling outputs and interactions with other cell-surface signalling molecules will yield fundamental new insights into how we may rationally design safer CAR-T cell therapies that do not compromise clinical efficacy.

## Figures and Tables

**Table 1:** Summary of FMC63 (CD19) CAR T cells in clinical trials with published clinical outcomes.

NCT ID	Tumour Type	No. of Patients	CAR Name	CAR Domain Structure					Gene Transfer Method	CAR-T Cell Dose	Ref
				ScFv	Hinge	TMD	Costim	Signal			
NCT02842138	FL	7	CD19-BBz(86)	FMC63	CD8α	CD8α	41BB	CD3ζ	lentivirus	3 × 10^8^–3.7 × 10^8^	[15]
NCT02842138	DLBCL	13	CD19-BBz(86)	FMC63	CD8α	CD8α	41BB	CD3ζ	lentivirus	6 × 10^6^–3.2 × 10^8^	[15]
NCT02030834	DLBCL, FL	28	CTL-019	FMC63	CD8α	CD8α	41BB	CD3ζ	lentivirus	3.1 × 10^6^–8.9 × 10^6^/kg	[39]
NCT02445248	DLBCL	111	CTL-019	FMC63	CD8α	CD8α	41BB	CD3ζ	lentivirus	1 × 10^7^–6 × 10^8^	[40]
NCT01626495 NCT01029366	B-ALL, T-ALL	30	CTL-019	FMC63	CD8α	CD8α	41BB	CD3ζ	lentivirus	1 × 10^7^–1 × 10^8^/kg	[30,41]
NCT02435849	B-ALL	75	CTL-019	FMC63	CD8α	CD8α	41BB	CD3ζ	lentivirus	3.1 × 10^6^/kg	[42]
NCT02631044	DLBCL	268	JCAR017	FMC63	CD8α	CD8α	41BB	CD3ζ	lentivirus	5 × 10^7^–1.5 × 10^8^	[43]
NCT01860937	B-ALL	25	19-28z	FMC63	CD28	CD28	CD28	CD3ζ	gamma retrovirus	1 × 10^6^–3 × 10^6^/kg	[44]
NCT01044069	B-ALL	53	19-28z	FMC63	CD28	CD28	CD28	CD3ζ	gamma retrovirus	1 × 10^6^–3 × 10^6^/kg	[23,45]
NCT00466531	CLL	16	19-28z	FMC63	CD28	CD28	CD28	CD3ζ	gamma retrovirus	2.6 × 10^6^–3.2 × 10^7^	[46]
NCT01840566	DLBCL, FL, MZL, MCL	15	19-28z	FMC63	CD28	CD28	CD28	CD3ζ	gamma retrovirus	5 × 10^6^–1 × 10^7^/kg	[47]
NCT02601313	MCL	68	KTE-X19	FMC63	CD28	CD28	CD28	CD3ζ	gamma retrovirus	2 × 10^6^/kg	[48]
NCT02348216	DLBCL, PMBCL, TFL	101	KTE-C19	FMC63	CD28	CD28	CD28	CD3ζ	gamma retrovirus	2 × 10^6^/kg	[49]
NCT00924326	DLBCL	17	CAR-19	FMC63	CD28	CD28	CD28	CD3ζ	gamma retrovirus	1 × 10^6^–2 × 10^6^/kg	[50]
NCT01593696	B-ALL	21	FMC63-28z	FMC63	CD28	CD28	CD28	CD3ζ	gamma retrovirus	3 × 10^4^–3 × 10^6^/kg	[51]
NCT02963038	B-ALL	10	SENL-B19	FMC63	CD28	CD28	41BB	CD3ζ	lentivirus	3 × 10^5^–1.6 × 10^6^/kg	[36]
NCT01865617	LBCL, FL, MCL	32	-	FMC63	IgG4	CD28	41BB	CD3ζ	lentivirus	2 × 10^5^–2 × 10^7^/kg	[37,38]

B-ALL, B cell acute lymphoblastic leukemia; CLL, chronic lymphocytic leukemia; DLBCL, diffuse large B cell lymphoma; FL, follicular lymphoma; MCL, mantle cell lymphoma; MZL, marginal zone lymphoma; PMBCL, primary mediastinal large B cell lymphoma.

**Table 2:** Effect of prognostic factors on complete response rate.

Prognostic Factor	No. of Patients	No. of Clinical Trials	Response Rate	*p*-Value
			Mean % [95% CI]	
Age:				0.7743
≤65	483	12	50.7% [39.1–62.3]	
>65	180	5	52.6% [24.0–81.1]	
Lymphodepletion:				0.4737
Yes	835	12	57.1% [46.9–67.4]	
No	13	3	42.9% [0–100]	
CAR-T cells infused:				0.2043
≤1 × 10^7^/kg	442	12	58.5% [47.0–70.0]	
>1 × 10^7^/kg	35	5	43.4% [5.8–81.0]	
Cancer type:				
B-ALL	184	5	72.3% [60.6–84.1]	
DLBCL	490	9	41.4% [32.6–50.2]	
FL	49	5	52.5% [30.2–74.7]	
MCL	73	3	22.2% [0–100]	

**Table 3:** CRS grading systems used in published FMC63 (CD19) CAR-T cell clinical trials.

NCT ID	CAR Name	CAR Domain Structure					No. of Patients	CRS Grading System	Neurotoxicity Grading System
		ScFv	Hinge	TM	Costim	Signal			
NCT02842138	CD19-BBz(86)	FMC63	CD8α	CD8α	41BB	CD3ζ	7	CTCAE v4.03 [57]	CTCAE v4.03 [57]
NCT02842138	CD19-BBz(86)	FMC63	CD8α	CD8α	41BB	CD3ζ	13	CTCAE v4.03 [57]	CTCAE v4.03 [57]
NCT02030834	CTL-019	FMC63	CD8α	CD8α	41BB	CD3ζ	28	Penn grading system [41]	CTCAE v3.0 [58]
NCT02445248	CTL-019	FMC63	CD8α	CD8α	41BB	CD3ζ	111	Penn grading system [41]	CTCAE v4.03 [57]
NCT01626495 NCT01029366	CTL-019	FMC63	CD8α	CD8α	41BB	CD3ζ	30	Penn grading system [41]	CTCAE v3.0 [58]
NCT02435849	CTL-019	FMC63	CD8α	CD8α	41BB	CD3ζ	75	MedDRA and CTCAE v4.03 [57]	MedDRA and CTCAE v4.03 [57]
NCT02631044	JCAR017	FMC63	CD8α	CD8α	41BB	CD3ζ	268	Lee et al. 2014 [60]	CTCAE v4.03 [57]
NCT01860937	19-28z	FMC63	CD28	CD28	CD28	CD3ζ	25	CTCAE v4.0	CTCAE v4.0
NCT01044069	19-28z	FMC63	CD28	CD28	CD28	CD3ζ	53	Lee et al. 2014 [60]	CTCAE v4.03 [57]
NCT00466531	19-28z	FMC63	CD28	CD28	CD28	CD3ζ	16	CTCAE v3.0 (<2009) [58] CTCAE v4.0 (>2009)	CTCAE v3.0 (<2009) [58] CTCAE v4.0 (>2009)
NCT01840566	19-28z	FMC63	CD28	CD28	CD28	CD3ζ	15	ASBMT	CTCAE v4.03 [57]
NCT02601313	KTE-X19	FMC63	CD28	CD28	CD28	CD3ζ	68	Lee et al. 2014 [60]	CTCAE v4.03 [57]
NCT02348216	KTE-C19	FMC63	CD28	CD28	CD28	CD3ζ	101	Lee et al. 2014 [60]	CTCAE v4.03 [57]
NCT00924326	CAR-19	FMC63	CD28	CD28	CD28	CD3ζ	17	CTCAE v3.0 [58]	CTCAE v3.0 [58]
NCT01593696	FMC63-28z	FMC63	CD28	CD28	CD28	CD3ζ	21	CTCAE v4.02	CTCAE v4.02
NCT02963038	SENL-B19	FMC63	CD28	CD28	41BB	CD3ζ	10	CTCAE v4.0	CTCAE v4.0
NCT01865617	-	FMC63	IgG4	CD28	41BB	CD3ζ	32	CTCAE v4.03 [57]	CTCAE v4.03 [57]

ASBMT, American Society for Blood and Marrow Transplantation Consensus Criteria; CTCAE, National Cancer Institute Common Terminology Criteria for Adverse Event; MedDRA, Medical Dictionary for Regulatory Authorities.

## Supplementary Materials

The following are available online at https://www.mdpi.com/2072-6694/13/1/38/s1, Table S1: Number and best clinical response of patients in each anti-CD19 CAR-T cell clinical trial, Table S2: Number and grade of patients diagnosed with CRS in each anti-CD19 CAR-T cell clinical trial, Table S3: Number and grade of patients diagnosed with neurotoxicity in each anti-CD19 CAR-T cell clinical trial.
