# Supplementary material for: The Influence of Chimeric Antigen Receptor Structural Domains on Clinical Outcomes and Associated Toxicities
Source: Cancers (Basel). 2020 Dec 25;13(1):38. doi: 10.3390/cancers13010038 (PMC7794933; doi:10.3390/cancers13010038)
Supplement: Supplementary file 1 [file cancers-13-00038-s001.pdf]

cancers

# Supplementary Materials: The Influence of Chimeric Antigen Receptor Structural Do-mains on Clinical Outcomes and Asso-ciated Toxicities

**Ashleigh S. Davey, Matthew E. Call and Melissa J. Call**

**Table S1.** Number and grade of patients diagnosed with CRS in each anti-CD19 CAR-T cell clinical trial.

| NCT ID | CAR Name | CAR domain structure | | | | | No. of pa-tients | Clinical Response (No. of patients) | | | | |
|---|---|---|---|---|---|---|---|---|---|---|---|---|
| | | ScFv | Hinge | TM | Costim | Signal | | SD | PD | PR | CR | NR |
| NCT02842138 | CD19-BBz(86) | FMC63 | CD8α | CD8α | 41BB | CD3ζ | 7 | 1 | 1 | 3 | 2 | 0 |
| NCT02842138 | CD19-BBz(86) | FMC63 | CD8α | CD8α | 41BB | CD3ζ | 13 | 1 | 6 | 4 | 5 | 0 |
| NCT02030834 | CTL-019 | FMC63 | CD8α | CD8α | 41BB | CD3ζ | 28 | - | - | 2 | 16 | 12 |
| NCT02445248 | CTL-019 | FMC63 | CD8α | CD8α | 41BB | CD3ζ | 93 | 14 | 24 | 11 | 37 | 7 |
| NCT01626495 NCT01029366 | CTL-019 | FMC63 | CD8α | CD8α | 41BB | CD3ζ | 30 | 3 | 0 | 3 | 27 | 0 |
| NCT02435849 | CTL-019 | FMC63 | CD8α | CD8α | 41BB | CD3ζ | 75 | 0 | 6 | 16 | 45 | 8 |
| NCT02631044 | JCAR017 | FMC63 | CD8α | CD8α | 41BB | CD3ζ | 255 | - | - | 51 | 135 | 69 |
| NCT01860937 | 19-28z | FMC63 | CD28 | CD28 | CD28 | CD3ζ | 25 | 0 | 6 | 0 | 18 | 1 |
| NCT01044069 | 19-28z | FMC63 | CD28 | CD28 | CD28 | CD3ζ | 53 | - | - | - | 44 | 1 |
| NCT00466531 | 19-28z | FMC63 | CD28 | CD28 | CD28 | CD3ζ | 16 | 7 | 5 | 0 | 3 | 1 |
| NCT01840566 | 19-28z | FMC63 | CD28 | CD28 | CD28 | CD3ζ | 15 | 2 | 4 | 0 | 8 | 1 |
| NCT02601313 | KTE-X19 | FMC63 | CD28 | CD28 | CD28 | CD3ζ | 68 | 2 | 2 | 16 | 40 | 8 |
| NCT02348216 | KTE-C19 | FMC63 | CD28 | CD28 | CD28 | CD3ζ | 101 | 11 | 5 | 28 | 55 | 2 |
| NCT00924326 | CAR-19 | FMC63 | CD28 | CD28 | CD28 | CD3ζ | 17 | 1 | 3 | 4 | 9 | 0 |
| NCT01593696 | FMC63-28z | FMC63 | CD28 | CD28 | CD28 | CD3ζ | 21 | 3 | 4 | 0 | 14 | 0 |
| NCT02963038 | SENL-B19 | FMC63 | CD28 | CD28 | 41BB | CD3ζ | 10 | 0 | 0 | 1 | 8 | 1 |
| NCT01865617 | - | FMC63 | IgG4 | CD28 | 41BB | CD3ζ | 32 | - | - | 9 | 10 | 13 |

CR, Complete Response; NR, No Record; PD, Progressive Disease; PR, Partial Response; SD, Stable Disease

**Table S2.** Number and grade of patients diagnosed with CRS in each anti-CD19 CAR-T cell clinical trial.

<table>
<tr><th rowspan="2">NCT ID</th><th rowspan="2">CAR Name</th><th colspan="5">CAR domain structure</th><th rowspan="2">No. of pa-tients</th><th colspan="5">CRS patient diagnosis (No. of patients)</th></tr>
<tr><th>ScFv</th><th>Hinge</th><th>TM</th><th>Costim</th><th>Signal</th><th>None</th><th>Grade 1</th><th>Grade 2</th><th>Grade 3</th><th>Grade 4+</th></tr>
<tr><td>NCT02842138</td><td>CD19-BBz(86)</td><td>FMC63</td><td>CD8α</td><td>CD8α</td><td>41BB</td><td>CD3ζ</td><td>7</td><td>6</td><td>1</td><td>0</td><td>0</td><td>0</td></tr>
<tr><td>NCT02842138</td><td>CD19-BBz(86)</td><td>FMC63</td><td>CD8α</td><td>CD8α</td><td>41BB</td><td>CD3ζ</td><td>16</td><td>11</td><td>5</td><td>0</td><td>0</td><td>0</td></tr>
<tr><td>NCT02030834</td><td>CTL-019</td><td>FMC63</td><td>CD8α</td><td>CD8α</td><td>41BB</td><td>CD3ζ</td><td>28</td><td>12</td><td>0</td><td>11</td><td>4</td><td>1</td></tr>
<tr><td>NCT02445248</td><td>CTL-019</td><td>FMC63</td><td>CD8α</td><td>CD8α</td><td>41BB</td><td>CD3ζ</td><td>111</td><td>47</td><td colspan="2">40</td><td>15</td><td>9</td></tr>
<tr><td>NCT01626495<br>NCT01029366</td><td>CTL-019</td><td>FMC63</td><td>CD8α</td><td>CD8α</td><td>41BB</td><td>CD3ζ</td><td>30</td><td>0</td><td colspan="2">22</td><td>8</td><td>0</td></tr>
<tr><td>NCT02435849</td><td>CTL-019</td><td>FMC63</td><td>CD8α</td><td>CD8α</td><td>41BB</td><td>CD3ζ</td><td>75</td><td>17</td><td colspan="2">23</td><td>16</td><td>19</td></tr>
<tr><td>NCT02631044</td><td>JCAR017</td><td>FMC63</td><td>CD8α</td><td>CD8α</td><td>41BB</td><td>CD3ζ</td><td>268</td><td>155</td><td colspan="2">107</td><td colspan="2">6</td></tr>
<tr><td>NCT01860937</td><td>19-28z</td><td>FMC63</td><td>CD28</td><td>CD28</td><td>CD28</td><td>CD3ζ</td><td>25</td><td>5</td><td colspan="2">16</td><td colspan="2">4</td></tr>
<tr><td>NCT01044069</td><td>19-28z</td><td>FMC63</td><td>CD28</td><td>CD28</td><td>CD28</td><td>CD3ζ</td><td>53</td><td>8</td><td colspan="2">31</td><td colspan="2">14</td></tr>
<tr><td>NCT00466531</td><td>19-28z</td><td>FMC63</td><td>CD28</td><td>CD28</td><td>CD28</td><td>CD3ζ</td><td>16</td><td>0</td><td>4</td><td>10</td><td>1</td><td>1</td></tr>
<tr><td>NCT01840566</td><td>19-28z</td><td>FMC63</td><td>CD28</td><td>CD28</td><td>CD28</td><td>CD3ζ</td><td>15</td><td>9</td><td>0</td><td>3</td><td>2</td><td>1</td></tr>
<tr><td>NCT02601313</td><td>KTE-X19</td><td>FMC63</td><td>CD28</td><td>CD28</td><td>CD28</td><td>CD3ζ</td><td>68</td><td>6</td><td>20</td><td>32</td><td>8</td><td>2</td></tr>
<tr><td>NCT02348216</td><td>KTE-C19</td><td>FMC63</td><td>CD28</td><td>CD28</td><td>CD28</td><td>CD3ζ</td><td>101</td><td>7</td><td>81</td><td>13</td><td>0</td><td>0</td></tr>
<tr><td>NCT00924326</td><td>CAR-19</td><td>FMC63</td><td>CD28</td><td>CD28</td><td>CD28</td><td>CD3ζ</td><td>17</td><td colspan="2">9</td><td>5</td><td>3</td><td>0</td></tr>
<tr><td>NCT01593696</td><td>FMC63-28z</td><td>FMC63</td><td>CD28</td><td>CD28</td><td>CD28</td><td>CD3ζ</td><td>21</td><td>5</td><td>8</td><td>2</td><td>3</td><td>3</td></tr>
<tr><td>NCT02963038</td><td>SENL-B19</td><td>FMC63</td><td>CD28</td><td>CD28</td><td>41BB</td><td>CD3ζ</td><td>10</td><td>0</td><td>5</td><td>1</td><td>3</td><td>1</td></tr>
<tr><td>NCT01865617</td><td>-</td><td>FMC63</td><td>IgG4</td><td>CD28</td><td>41BB</td><td>CD3ζ</td><td>32</td><td>12</td><td>5</td><td>6</td><td>6</td><td>3</td></tr>
</table>

**Table S3.** Number and grade of patients diagnosed with neurotoxicity in each anti-CD19 CAR-T cell clinical trial.

<table>
<tr><th rowspan="2">NCT ID</th><th rowspan="2">CAR Name</th><th colspan="5">CAR domain structure</th><th rowspan="2">No. of patients</th><th colspan="5">Neurotoxicity patient diagnosis (No. of patients)</th></tr>
<tr><td>ScFv</td><td>Hinge</td><td>TM</td><td>Costim</td><td>Signal</td><td>None</td><td>Grade 1</td><td>Grade 2</td><td>Grade 3</td><td>Grade 4+</td></tr>
<tr><td>NCT02842138</td><td>CD19-BBz(86)</td><td>FMC63</td><td>CD8α</td><td>CD8α</td><td>41BB</td><td>CD3ζ</td><td>7</td><td>7</td><td>0</td><td>0</td><td>0</td><td>0</td></tr>
<tr><td>NCT02842138</td><td>CD19-BBz(86)</td><td>FMC63</td><td>CD8α</td><td>CD8α</td><td>41BB</td><td>CD3ζ</td><td>16</td><td>16</td><td>0</td><td>0</td><td>0</td><td>0</td></tr>
<tr><td>NCT02030834</td><td>CTL-019</td><td>FMC63</td><td>CD8α</td><td>CD8α</td><td>41BB</td><td>CD3ζ</td><td>28</td><td>17</td><td>4</td><td>4</td><td>1</td><td>2</td></tr>
<tr><td>NCT02445248</td><td>CTL-019</td><td>FMC63</td><td>CD8α</td><td>CD8α</td><td>41BB</td><td>CD3ζ</td><td>111</td><td>88</td><td colspan="2">10</td><td>8</td><td>5</td></tr>
<tr><td>NCT01626495<br>NCT01029366</td><td>CTL-019</td><td>FMC63</td><td>CD8α</td><td>CD8α</td><td>41BB</td><td>CD3ζ</td><td>30</td><td>-</td><td colspan="2">-</td><td>-</td><td>-</td></tr>
<tr><td>NCT02435849</td><td>CTL-019</td><td>FMC63</td><td>CD8α</td><td>CD8α</td><td>41BB</td><td>CD3ζ</td><td>75</td><td>45</td><td colspan="2">20</td><td>10</td><td>0</td></tr>
<tr><td>NCT02631044</td><td>JCAR017</td><td>FMC63</td><td>CD8α</td><td>CD8α</td><td>41BB</td><td>CD3ζ</td><td>268</td><td>188</td><td colspan="2">53</td><td colspan="2">27</td></tr>
<tr><td>NCT01860937</td><td>19-28z</td><td>FMC63</td><td>CD28</td><td>CD28</td><td>CD28</td><td>CD3ζ</td><td>25</td><td>7</td><td colspan="2">11</td><td colspan="2">7</td></tr>
<tr><td>NCT01044069</td><td>19-28z</td><td>FMC63</td><td>CD28</td><td>CD28</td><td>CD28</td><td>CD3ζ</td><td>53</td><td colspan="2">30</td><td>1</td><td colspan="2">19</td></tr>
<tr><td>NCT00466531</td><td>19-28z</td><td>FMC63</td><td>CD28</td><td>CD28</td><td>CD28</td><td>CD3ζ</td><td>16</td><td>10</td><td>3</td><td>2</td><td>1</td><td>0</td></tr>
<tr><td>NCT01840566</td><td>19-28z</td><td>FMC63</td><td>CD28</td><td>CD28</td><td>CD28</td><td>CD3ζ</td><td>15</td><td>5</td><td>0</td><td>1</td><td>5</td><td>4</td></tr>
<tr><td>NCT02601313</td><td>KTE-X19</td><td>FMC63</td><td>CD28</td><td>CD28</td><td>CD28</td><td>CD3ζ</td><td>68</td><td>25</td><td>13</td><td>9</td><td>15</td><td>6</td></tr>
<tr><td>NCT02348216</td><td>KTE-C19</td><td>FMC63</td><td>CD28</td><td>CD28</td><td>CD28</td><td>CD3ζ</td><td>101</td><td>36</td><td>37</td><td>28</td><td>0</td><td>36</td></tr>
<tr><td>NCT00924326</td><td>CAR-19</td><td>FMC63</td><td>CD28</td><td>CD28</td><td>CD28</td><td>CD3ζ</td><td>17</td><td colspan="2">5</td><td>5</td><td colspan="2">7</td></tr>
<tr><td>NCT01593696</td><td>FMC63-28z</td><td>FMC63</td><td>CD28</td><td>CD28</td><td>CD28</td><td>CD3ζ</td><td>21</td><td>15</td><td>2</td><td>3</td><td>1</td><td>0</td></tr>
<tr><td>NCT02963038</td><td>SENL-B19</td><td>FMC63</td><td>CD28</td><td>CD28</td><td>41BB</td><td>CD3ζ</td><td>10</td><td>4</td><td>0</td><td>0</td><td>5</td><td>1</td></tr>
<tr><td>NCT01865617</td><td>-</td><td>FMC63</td><td>IgG4</td><td>CD28</td><td>41BB</td><td>CD3ζ</td><td>32</td><td>24</td><td>0</td><td>0</td><td>6</td><td>2</td></tr>
</table>
